# Supplementary material for: Receptor-Mediated AKT/PI3K Signalling and Behavioural Alterations in Zebrafish Larvae Reveal Association between Schizophrenia and Opioid Use Disorder
Source: Int J Mol Sci. 2022 Apr 25;23(9):4715. doi: 10.3390/ijms23094715 (PMC9104710; doi:10.3390/ijms23094715)
Supplement: Supplementary file 1 [file ijms-23-04715-s001.zip › ijms-1669189-supplementary.pdf]

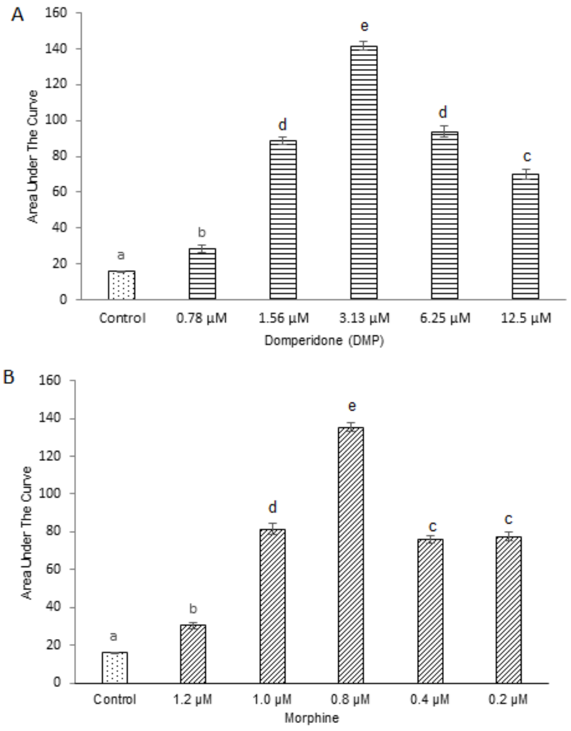

Figure S1. Startle habituation response of zebrafish larvae. Comparison of AUC of the two treatment groups at different concentrations. Data shown in (**A**) and (**B**) are mean ± SEM (n = 15/concentration). Different lowercase letters indicate statistically significant values, $p < 0.05$.
